# Legume-Cereal Intercropping Improves Forage Yield, Quality and Degradability

**DOI:** 10.1371/journal.pone.0144813

**Published:** 2015-12-16

**Authors:** Jie Zhang, Binjie Yin, Yuhuai Xie, Jing. Li, Zaibin Yang, Guiguo Zhang

**Affiliations:** 1 College of Animal Sciences and Technology, Shandong Agricultural University, Tai-an, Shandong 271018, P. R. China; 2 Department of Biochemistry and Molecular Biology, College of Life Sciences, Peking University, Beijing 100871, P. R. China; Northwest A&F University, CHINA

## Abstract

Intercropping legume with cereal is an extensively applied planting pattern in crop cultivation. However, forage potential and the degradability of harvested mixtures from intercropping system remain unclear. To investigate the feasibility of applying an intercropping system as a forage supply source to ruminants, two consecutive experiments (experiments 1 and 2) involving a field cultivation trial and a subsequent *in vivo* degradable experiment were conducted to determine the forage production performance and the ruminally degradable characteristics of a harvested mixture from an alfalfa/corn-rye intercropping system. In experiment 1, the intercropping system was established by alternating alfalfa and corn or rye with a row ratio of 5:2. Dry matter (DM) and nutrient yields were determined. In experiment 2, forages harvested from the different treatments were used as feedstuff to identify nutrient degradation kinetics and distribution of components between the rapidly degradable (*a*), potentially degradable (*b*) and the degradation rate constant (*c*) of ‘*b*’ fraction by *in sacco* method in Small-Tail Han wether Sheep. The intercropping system of alfalfa and corn-rye provided higher forage production performance with net increases of 9.52% and 34.81% in DM yield, 42.13% and 16.74% in crude protein (CP) yield, 25.94% and 69.99% in degradable DM yield, and 16.96% and 5.50% in degradable CP yield than rotation and alfalfa sole cropping systems, respectively. In addition, the harvest mixture from intercropping system also had greater ‘*a*’ fraction, ‘*b*’ fraction, ‘*c*’ values, and effective degradability (*E* value) of DM and CP than corn or rye hay harvested from rotation system. After 48-h exposure to rumen microbes, intercropping harvest materials were degraded to a higher extent than separately degraded crop stems from the sole system as indicated by visual microscopic examination with more tissues disappeared. Thus, the intercropping of alfalfa and corn-rye exhibited a greater forage production potential, and could be applied as forage supply source for ruminants. The improved effective degradability of harvest mixture material could be attributed to greater degradable components involving the rapidly degradable fractions (*a*), potentially degradable (*b*) fractions, and degradable rate constant (c), than that of corn and rye hay.

## Introduction

In many farming areas of the world, the continuous reduction of arable land has decreased the available land area for forage production [1,2]. Crop residues, which lack rumen-degradable nutrients [3,4], have been recommended as the main roughage resource in intensive ruminant farming systems [5]. However, deficient superior forage is the main restrictive factor in herbivorous livestock industry in those regions [6,7,8]. This deficiency, including unbalanced seasonal forage distribution, can be resolved by purchasing and storing high quality roughage feed such as hay or silage for ruminants [9,10]. Despite the advantage of this practice, production costs are likely increased and farm profitability is reduced. Thus, feasible methods should be established to satisfy roughage requirement for rapid and sustainable development of ruminant husbandry [6,11,12].

Intercropping exhibits a greater forage production performance than sole cropping and is a feasible option for forage production [13,14,15]. Corn (*Zea mays* L.)-rye (*Secale cereale* L.) rotation and their respective monocultures are traditional cultivation practices in the main grain and forage production system in farming areas worldwide [16], especially in some areas that are limited to only one cropping season annually due to temperature and water constraints. Furthermore, the corn-based intercropping with alfalfa (*Medicago sativa* L.) [17,18], faba bean (*Vicia faba* L.) [19,20], soybean (*Glycine max* L. Merr.), and wheat (*Triticum aestivum* L.) [21], have been documented a yield advantage compared with sole cropping of corn or legumes. Previous studies on forage degradability also showed that the degradable rate and extent of forage tissues were related to the inherent degradable characteristics (e.g. the content of rapidly soluble fraction, the content of slowly degradable fraction and its rate constant (h^-1^) of the degradation); these parameters can be investigated on the basis of anatomical structure variation [22,23].

Although advantages of biomass yields of intercropping system with two crops species have been extensively investigated, further studies have yet to evaluate whether intercropping alfalfa with corn-rye rotation system is feasible when considering the degradability or utilization of harvest mixture from intercropping system. In addition, degradation characteristics of mixture harvested from intercropping systems also remain unclear. In the present study, rye was intercropped with alfalfa after corn was harvested on the basis of existing alfalfa/corn strip intercropping systems to improve the land utilization and avoid the land idle after corn was harvest. This study aimed to (1) determine the biomass and nutrient yields of alfalfa/corn-rye intercropping system, (2) explore the degradable characteristics of mixture by *in sacco* method, and (3) examine changes in the anatomical structure of crop stem before and after degradation occurs.

## Materials and Methods

### Plant materials and experimental design

Field experiments were conducted at the Chinese National Huang-Huai-Hai Regional Corn Technology Innovation Centre (36°09' N, 117°09' E, 134 m asl), on a brown loam soil (fine, mixed, super-active, mesic). Organic matter, total N, Olsen P and K in the upper 20cm of soil in the experimental field were 8.1%, 3.45 g/kg, 31.6, and 78.2 mg/kg, respectively. The centre, managed by Shandong Agricultural University, is specially used for field cultivation trials. The region is characterized with a temperate, semiarid and continental climate. Annual sunshine time is 2,611 h. The frost-free period is approximately 200 days and precipitation is approximately 650 mm.

**Fig 1:**
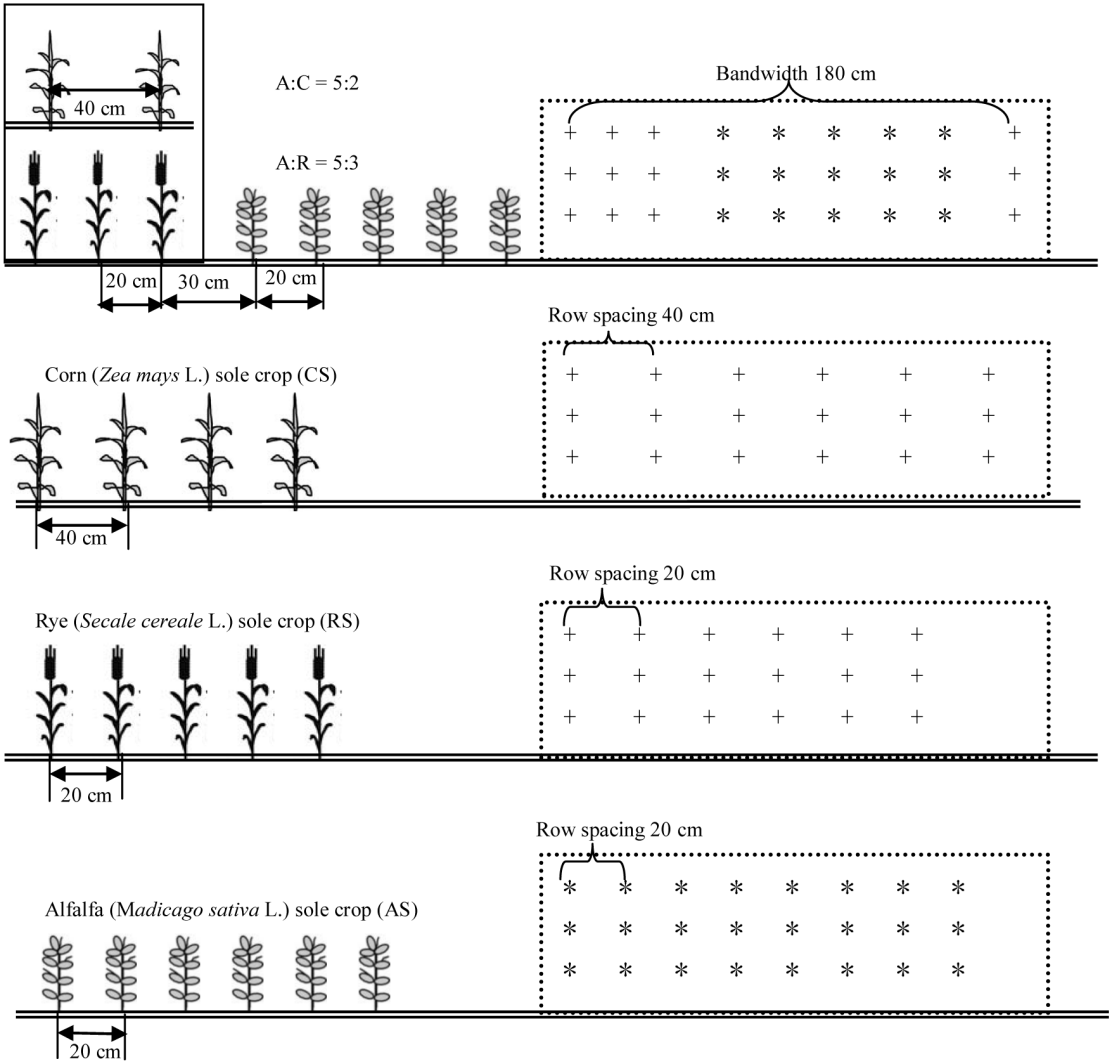
Diagram of different intercropping patterns between alfalfa (A, *), corn (C, +) and rye (R, +). Distances between rows (cm) are indicated.

In the present field experiment, three cultivation treatments involving the intercropping of alfalfa (*Medicago sativa* L. *cv*. ‘Algonquin’)/corn (*Zea mays* L. *cv*. ‘Zhengdan 958’)-rye (*Secale cereale* L. *cv*. ‘Lumai1’), rotation of corn-rye, and sole crop alfalfa were arranged following a randomized complete block design with 3 replications. Based on the previous studies where 5 rows alfalfa were alternatively intercropped with 2 rows corn exhibiting a higher yield advantage compared to respective sole cropping [14], an intercropping system with an alfalfa:corn row ratio of 5:2was established in May 20, 2011 in a 23.4m^2^ plot. The rotation of corn-rye and sole crop alfalfa were established in the same area plots at the same time. In the second year (2012) of the establishment of the alfalfa/corn intercropping system, rye was introduced to the intercropping system after corn was harvested and grown with alfalfa from October to May of the succeeding year (2013) in intercropping plots. Three rows of rye were planted with a row spacing of 20 cm between two alfalfa bands (Fig 1). At the first year of the establishment of intercropping system, all plots were given a basal application of N at 225 kg ha^-1^ as urea and P were applied at 33 kg ha^-1^ as calcium superphosphate. In the following years, no fertilizes were applied to any of the plots during the experiment. Both the N and P fertilizers were evenly broadcast and incorporated into the top 20 cm of the soil prior to sowing. The general irrigation and pesticide practices were employed in proper time during the growing season to prevent the water stress and insect pest occurrence. Climatic information for the duration of the experiment is shown in Table 1, and no major natural disasters occurred.

**Table 1:** Weather conditions for the duration of the experiment.

Item		Months											
		1	2	3	4	5	6	7	8	9	10	11	12
2012 (year)													
Rainfall (mm/month)		6.5	8.6	18.2	25.8	44.2	90.6	218.2	157.7	67.4	35.3	18.4	9.2
Air temperature (°C)	Mean minimum	-7.4	5.2	1	7.6	13.3	19.8	22.3	21.2	15.4	9.6	-1.8	-5.6
	Mean maximum	4.4	6.5	13.2	20	26.3	31.6	31.8	31.1	32.9	29.3	18.8	6.3
2013 (year)													
Rainfall (mm/month)		4.2	7.1	16.7	27.2	68.8	118.5	203.4	124	75.6	29.3	26.3	12.3
Air temperature (°C)	Mean minimum	-7.1	-8.2	-1.2	6.8	7.7	16.1	17.9	17.7	11.6	8.6	-3.2	-6.2
	Mean maximum	8.3	10.6	17.1	24	27.3	31.3	34.1	32.6	31.1	29.9	24.7	8.5

In the second year of intercropping system establishment (2012), the biomass and nutrient yields were determined. Alfalfa was harvested at one-tenth bloom (the early blooming stage) to a 5-cm stubble height without seriously reducing plant vigour and stand life. In our study, alfalfa was reaped 5m of each strip at early blooming stage for determining DM yields, and harvested 4 times in 2012, and 2 times in the first half of 2013 (to accumulate the alfalfa yield before rye harvest). Grain and stalk of corn were harvested separately at physiological maturity by cutting 10 plants randomly from each plot. Rye was harvested 5 m of each strip in heading stages for calculating biomass yield.

The harvested samples were oven-dried at 65°C until the weight was constant to measure the content of DM and calculated the biomass yield of all treatments [14,17].

### In sacco incubation

This study was carried out in strict accordance with the recommendations in the Guide for the Care and Use of Laboratory Animals of Shandong Agricultural University Animal Nutrition Research Institute. The protocol was approved by the Committee on the Ethics of Shandong Agricultural University (Approval Number: S20130058). All efforts were made to minimize suffering.

A degradability study was conducted through *in sacco* incubation in 3 rumen-fistulated mature ‘Small-Tail Han’ wether sheep (average body weight 45 ± 5 kg) to evaluate the degradability of separate alfalfa, rye, corn (stem + grain) from sole cropping, and the mixture from intercropping harvest. The sheep were fed with a total mixture ratio containing 40% alfalfa, 40% rye hay and 20% concentrate. Diet and water were provided *ad libitum*.

Oven-dried (65°C) corn (stem + grain), rye, and alfalfa hay were ground to pass 1-mm sieve. The ground samples were weighed to approximately 8 g, and placed individually in N-free polyester bags (10cm×9cm with a pore size of 50 ± 15 μm; Ankom Technology, Macedon, NY, USA). The mixture from intercropping contained the three materials following the field biomass yield proportion (rye hay: corn stalk: corn grain: alfalfa hay = 1:1.5:1.5:4) and a weight of 8 g.

The N-free polyester bags filled into samples were heat-sealed. Then, 8 bags (2 bags for each sample) were placed in a weighted (200 g) lingerie bag (25 cm×15cm in size) and warmed in water (39°C) for 20 min; afterward, the lingerie bags were placed in the ventral rumen and incubated for 0, 2, 4, 6, 12, 16, 24, and 72 h. For each incubation time, one lingerie bag containing four duplicate samples was placed into each of the three sheep rumen, for a total of six replicated samples per treatment. Incubations were run consecutively.

The non-incubation bags (0 h) were not incubated in the rumen, but kept under water at 39°C for additional 20 min, and then washed with tap water for 15 min in a washing machine where rinsing was interrupted thrice by spinning.

After the appropriate incubation time, six duplicate bags were removed at each incubation time for each sample and immediately placed in iced water for 5 min. Subsequently, the bags were rinsed followed by a rinsing in the washing machine with spinning. All of the washed bags were oven-dried at 65°C to determine residue DM, then the dried residues from bags were collected and ground to pass 1-mm sieve for chemical analysis.

### Microscopic analysis

Based on the differences in degradability among samples from sole crop and intercropping systems, specific internodes (the fourth elongated internodes above the soil surface for corn and rye [23], and the seventh internodes for alfalfa [22]) preserved in 50g/100g ethanol were selected and microscopically evaluated to visualize degradable sections. Twenty slices of each crop stalk with cross-sections of 100 μm in thickness were prepared using a sliding-type microtome (Leica SM 2400, GMI, Inc., USA) and stored in 50g/100g ethanol. Ten randomly chosen sections of each crop were used to observe the anatomic structure of forage stem and the remaining 10 sections were degraded by *in vitro* method for 48 h incubation [23,24].

The *in vitro* trial were designed to 3 incubation groups corresponding to the 3 harvest materials from the field trial, i.e. sole crop corn stalk, sole crop rye stalk sections, and mixed samples from intercropping system composed of corn stalk, rye stalk, alfalfa stem, and corn grain (0.5 g) ground to pass 1.0-mm screen. The section samples were incubated in 150 mL serum bottle with 100 mL of a 250 mg/g rumen fluid:buffer mixture [23]. These bottles were placed in an incubator with 39°C water bath and periodically agitated under continuous flushing with CO_2_ for 48 h incubation. Rumen fluid was collected from a rumen-fistulated small-tail sheep used in *in sacco* experiment; the sheep were fed and treated in the same manner. After incubation was completed, the sections were gently rinsed with tap water and then stored in 500 mg/g ethanol for microscopic evaluation. The sections were stained with safranine for 1 min, rinsed gently with running tap water, and then stained using fast green for 20 s; excess solution was removed. Stained sections were examined for tissue degradation degree by light microscopy (Diagnostic Instruments, Inc., Sterling Heights, MI) and images were collected using a spot digital camera (Nikon, D7000).

### Calculation of degradation kinetics and effective degradability

The time-course disappearance for DM, crude protein (CP) and neutral detergent fiber (NDF) were used to fit the *in sacco* degradation kinetic parameters using non-linear (NLIN) procedure of SAS (2003) following the formula below [25,26].

$$p(t)=a+b[1-e^{-c(t-L)}]$$ (1)

where *p* (*t*) is the proportion of feed degraded at the incubation time ‘*t*’, ‘*a’* is the rapidly soluble fraction (the proportion that can be washed out of the bags at 0 h), ‘*b’* is the degradable but insoluble fraction, ‘*c*’ is the rate constant (h^-1^) of the degradation of fraction ‘*b*’, and ‘*L*’ is the lag time (h) before disappearance began. Potentially degradable fraction ‘*d*’ was calculated as the sum of ‘*a’* and ‘*b’*.

Furthermore, the effective degradability (*E*) of DM, CP, and NDF was obtained as the following equation:

E=a+bc/(c+k) (2)

where ‘k’ is the fractional passage rate assumed to be 0.06 for forage [27].

### Chemical component analysis

The same nutrient component in feed or *in sacco* residues was determined following the same procedure. Crude protein was determined by applying the micro-Kjeldahl method [28]. The NDF were measured according to a previously described procedure [29].

### Statistical analysis

Data on DM yield, nutrient yields, and degradation kinetics were subjected to one-way ANOVA using the GLM procedure of SAS version 9.0. The effect of cultivation patterns on yields was determined by the preplanned contrast (intercropping vs. others). Difference was declared significant when *P* < 0.05. Means of the feed degradation kinetics between treatments were compared with Duncan’s multiple-comparison range test at 5% level.

## Results

### The DM and nutrient yields

Highly significant (*P*<0.001) differences in DM, CP, and NDF yields among different cultivation patterns were observed (Table 2). The intercropping system exhibited higher (*P*≤0.002) DM and CP yields, and lower (*P* = 0.001) NDF yield than the rotation of corn and rye. Compared with sole crop of alfalfa, the intercropping system considerably improved (*P*≤0.002) DM, CP, and NDF yields. However, regardless of intercropping or sole cropping, the contents of CP and NDF for the same crop were always similar (*P*>0.05).

**Table 2:** Dry matter and nutrient yields (kg/ha) of different cultivation patterns.

Treatments		DM Yield (kg/ha)	Nutrient Content (%)		Nutrient Yield (kg/ha)	
			CP	NDF	CP	NDF
Intercropping	corn	11952	8.89	45.4	1062	5427
	rye	4750	10.8	64.2	505	3050
	alfalfa	17701	19.3	37.5	3422	6631
	summed	34403	/	/	4989	15108
Rotation	corn	23471	8.81	44.9	2068	10538
	rye	7656	9.98	64.1	764	4907
	summed	31127	/	/	2887	15574
Sole crop	alfalfa	22428	18.5	37.4	4154	8402
SEM*		153.9	0.71	0.27	26.1	60.3
ANOVA						
Main effects		<0.001	<0.001	<0.001	<0.001	<0.001
Intercropping.*vs*.	rotation	/	/	/	/	/
	corn	/	0.590	0.945	/	/
	rye	/	0.419	0.957	/	/
	summed	0.002	/	/	<0.001	0.001
Intercropping.*vs*.	sole crop alfalfa	<0.001	0.797	0.872	0.002	<0.001

* The SEM values represent the overall standard error of means in each column.

### Degradability and degradable nutrient yields at 48 h incubation

The degradability of DM, CP, and NDF at 48 h is shown in Table 3. The nutrient degradability of intercropping mixture material was markedly higher (*P*<0.01) than that of corn or rye except for NDF of corn (*P* = 0.167), and lower (*P*<0.01) than that of alfalfa. Based on 48 h degradability, degradable DM and nutrient yields were significantly different (*P*<0.001) among the present cultivation patterns. The degradable DM and CP yields of the intercropping system were substantially greater (*P*<0.01) than those of sole cropping and rotation cropping. However, the degradable NDF yield of the intercropping system was similar (*P* = 0.087) to that of rotation cropping and higher (*P*<0.001) than that of the sole cropping of alfalfa.

**Table 3:** The 48-h degradability and yields of degradable dry matter (DM), crude protein (CP)and neutral detergent fiber (NDF) based on 48-h degradability.

Treatments		Degradability (%)			Degradable nutrient yields (kg/ha)		
		DM	CP	NDF	DM	CP	NDF
Intercropping	mixture#	58.8	85	65.6	20218	4239	9907
Rotation	corn	52.6	43.9	63.6	12343	913	6776
	rye	34.4	44.4	56.7	2630	358	2788
	summed	/	/	/	14973	1272	9564
Sole crop	alfalfa	74.9	96.4	78.3	16789	4006	6579
SEM*		0.56	0.48	0.66	171.4	25.7	82.8
ANOVA							
Main effects		<0.001	<0.001	<0.001	<0.001	<0.001	<0.001
Intercropping.*vs*.	rotation						
	corn	<0.001	<0.001	0.167	/	/	/
	rye	<0.001	<0.001	<0.001	/	/	/
	summed	/	/	/	<0.001	<0.001	0.087
Intercropping.*vs*.	sole crop alfalfa	<0.001	<0.001	<0.001	<0.001	0.013	<0.001

^#^Mixture was the harvest material from intercropping system composed of corn (stalk + grain), rye, and alfalfa according to the actual yield in field. i.e. rye hay: corn stalk: corn grain: alfalfa hay = 1:1.5:1.5:4.

* The SEM values represent the overall standard error of means in each column.

### In sacco degradation kinetics

Table 4 presents the *in sacco* degradation parameters of DM, CP, and NDF determined in the test feeds. The rapidly degradable DM of intercropping harvest material in the ‘*a*’ fraction was greater (*P*<0.001) than that of corn or rye stems subjected to rotation cropping and less than that of alfalfa subjected to sole cropping. The ‘*b*’ fraction (slowly degradable) and the ‘*c*’ value (degradation rate of ‘*b*’ fraction) of harvest materials subjected to intercropping were not less than those of materials subjected to rotation cropping (*P* = 0.011) or alfalfa subjected to sole cropping (*P* = 0.071). However, harvest mixture from intercropping exhibited higher degradable potential (‘*d*’ values), effective degradability (‘*E*’ values), and less lag time (‘*L*’ value) than that from rotation cropping. Alfalfa subjected to sole cropping showed the numerically greatest ‘d’, ‘*E*’, and ‘*L*’ values among the tested feeds.

**Table 4:** The *in sacco* degradation kinetics* of different cultivation patterns.

	Test feedstuffs				SEM	Effects *P*-values
	Rye	Corn	Mixture^#^	Alfalfa		
DM						
a	12.0d^†^	25.6c	28.6b	49.2a	0.84	<0.001
b	31.6b	34.9a	37.4a	33.0ab	1.38	0.011
c	0.035b	0.043ab	0.038ab	0.045a	0.0025	0.071
d	43.6d	60.4c	65.9b	80.2a	0.76	<0.001
L	0.94c	1.38b	0.16d	6.75a	0.386	<0.001
E	37.3d	46.0c	49.3b	66.8a	0.38	<0.001
CP						
a	23.8c	20.9d	69.5b	75.6a	0.24	<0.001
b	23.8b	28.1a	17.9d	21.9c	0.3	<0.001
c	0.065a	0.040c	0.052b	0.054b	0.0017	<0.001
d	47.6d	49.0c	87.4b	97.5a	0.41	<0.001
L	3.02a	2.63a	1.88b	1.41c	0.126	<0.001
E	40.0d	36.9c	80.9b	89.7a	0.12	<0.001
NDF						
a	23.5c	40.2b	37.8b	48.6a	1.44	<0.001
b	33.3a	26.3b	36.3a	33.8a	1.61	0.012
c	0.018d	0.093a	0.036c	0.050b	0.7464	<0.001
d	56.9d	66.4c	74.1b	82.4a	1.27	<0.001
L	2.06c	2.89b	2.29b	6.87a	0.774	0.002
E	52.1d	56.0c	57.5b	69.8a	0.5	<0.001

*a, soluble fraction (%); b, degradable but insoluble fraction (%); c, rate constant (%/h) of degradation of fraction b; d = a+ b, the potential degradable fraction (%); L, lag time (h) and E, effective degradability calculated with a passage rate (k) of 6%/h.

^†^Different letters in the same row indicate significant differences (*P*< 0.05).

^#^Mixture was the harvest material from intercropping system composed of corn (stalk + grain), rye, and alfalfa according to the actual yield in field. i.e. rye hay: corn stalk: corn grain: alfalfa hay = 1:1.5:1.5:4.

The rapidly degradable CP (*a*), potential degradable CP (*d*), and effective degradability (*E*) of CP of the harvest material subjected to intercropping were higher than those of cereals subjected to sole cropping and less than those of alfalfa hay subjected to sole cropping. While the lowest slowly degradable fraction (*b*) was observed in the mixture subjected to intercropping compared with cereals or alfalfa hay subjected to sole cropping. The lag time (*L*) of CP of the mixture subjected to intercropping was shorter than that of cereals subjected to sole cropping but longer than that of alfalfa hay subjected to sole cropping. The degradation rate (*c*) of slowly degradable CP was similar to that of the mixture subjected to intercropping and alfalfa hay but less than that of cereals subjected to sole cropping.

The rapidly degradable fraction (*a*), potential degradable fraction (*d*), and effective degradability (*E*) of NDF were different among the test feeds, with the highest in alfalfa hay, followed by intercropping mixture, and the lowest in sole crop cereals. The degradation rate (*c*) of NDF was lower (*P*<0.001) in mixture subjected to intercropping compared to alfalfa hay and corn subjected to sole cropping. The lag time of NDF in the mixture subjected to intercropping averaged 2.29 h; this parameter was shorter than that of whole corn (2.89 h; *P* < 0.05) and alfalfa hay (6.86 h; *P* <0.05) but higher than that of rye hay (2.06 h; *P* < 0.05).

### Comparison of anatomical structures of forage stalk before and after degradation in rumen

**Fig 2:**
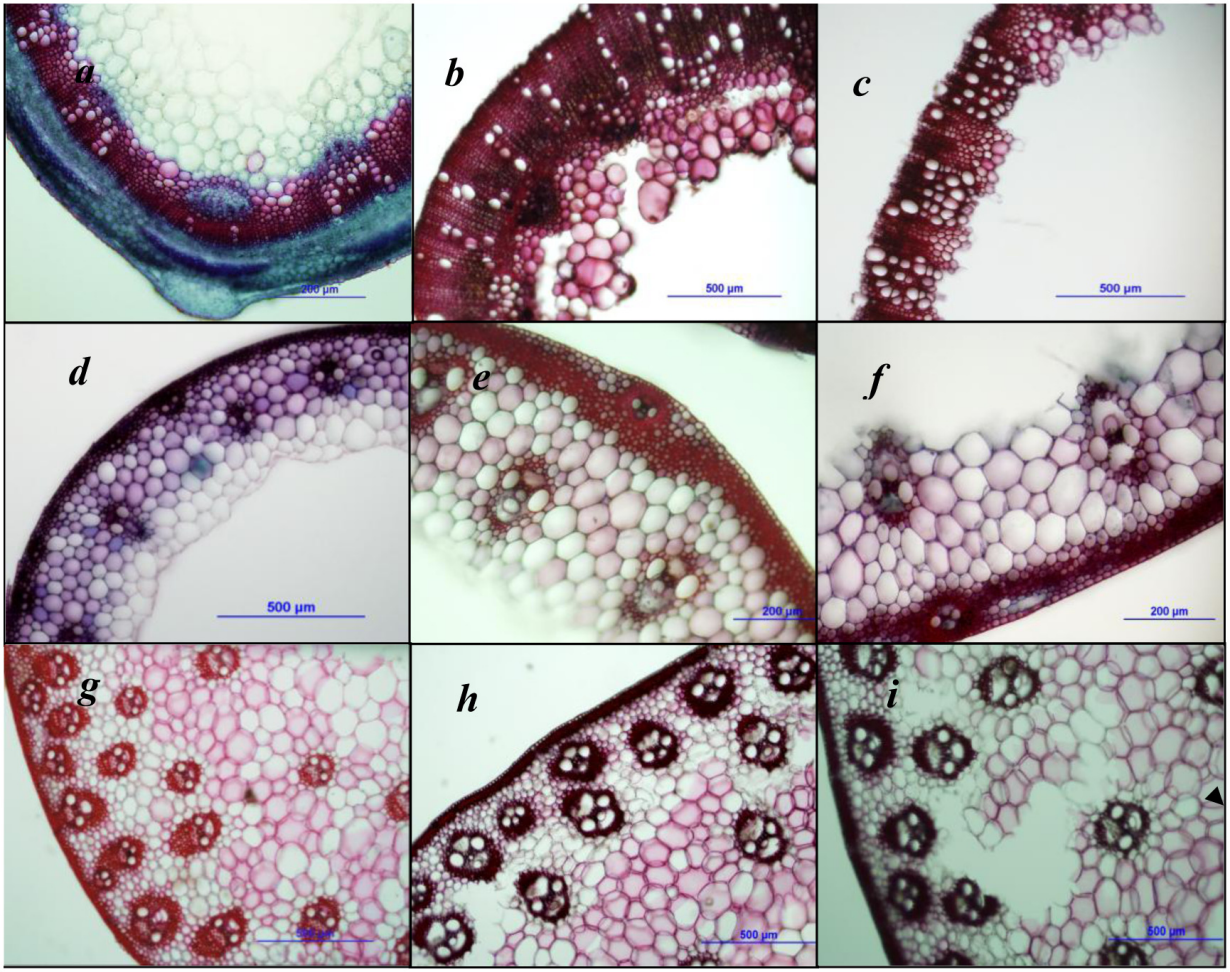
Transverse sections of alfalfa, rye and corn stems before and after degradation. *a*. the anatomic structure of alfalfa stem (the cross-section of the seventh internode); *b*. the anatomic structure of rumen-degraded alfalfa stem subjected to sole cropping; *c*. the anatomic structure of intercropping alfalfa stem degraded together with rye and corn stem in rumen; *d*. the anatomic structure of rye stem (the cross-section of the fourth internode); *e*. the anatomic structure of rumen-degraded rye stem subjected to sole cropping; *f*. the anatomic structure of intercropping rye stem degraded with alfalfa and corn stem in rumen; *g*. the anatomic structure of corn stalk (the cross-section of the fourth internode); *h*. the anatomic structure of rumen-degraded corn stalk subjected to sole cropping; *i*. the anatomic structure of intercropping corn stalk degraded with rye and alfalfa stem in rumen.

Fig 2 showed an overview of the stalk sections that typically remain after 48-h degradation by rumen microbes. The anatomical structure of the samples was greatly altered compared with that of samples before degradation occurred.

Lignified alfalfa vascular bundles formed a continuous ring-like structure around the stem, and parenchyma cell walls in the pith were very thin and non-lignified (Fig 2*a*). Vascular bundles of corn and rye stalks discretely distributed throughout the parenchyma of the stalks, as observed in the cross-sections (Fig 2*d* and 2*g*). In the cross-section of the rye stem, parenchyma cells adjacent to the epidermis were very small, whereas those around the center of the pith were relatively large. Discrete vascular bundles were also separated by regions of parenchyma cells. The primary phloem and xylem in the vascular bundles were prominent but lack in fascicular cambium (Fig 2*d*).

Degradation studies with mirror sections have revealed that the epidermis and collenchyma cells of the alfalfa stem subjected to sole cropping (separately degraded) were completely degraded, but the parenchyma cells in the pith and the fascicular cambium, as well as the phloem close to the xylem ring structure, were only partly degraded after 48-h exposure to rumen microbes (Fig 2*b*). Nevertheless, the sections of alfalfa stem subjected to intercropping were degraded together with corn and rye stalks; these sections were degraded to a greater extent than separately degraded alfalfa stems, and all of the tissues except the xylem ring structure were almost completely degraded by microbial activity (Fig 2*c*).

After 48-h exposure to rumen microbes, it appeared that only the parenchyma cells close to the pith and some of the epidermis cells from rye stems subjected to sole cropping were degraded. However, the vascular bundles and the surrounding parenchyma cells were completely non-degradable (Fig 2e). While for the intercropping rye stems degraded with alfalfa and corn stalks, the parenchyma cells neighboring the pith were completely degraded, and the epidermis region showed some holes; moreover, the parenchyma around the vascular bundles were also degraded to a greater extent compared with rye stalk subjected to sole cropping (Fig 2*f*).

After corn stalk subjected to sole cropping was exposed to rumen microbes for 48h, only small amount of parenchyma cells inlayed in the vascular bundles were degraded (Fig 2*h*). By contrast, for intercropping corn stalk sections degraded with alfalfa and rye stems, a large number of parenchyma cells surrounding vascular bundles were degraded, and more holes were observed in the degraded sections (Fig 2*i*). In the present trial, parenchyma cells located in the inner central pith region of the cross-sections of corn stalk were poorly degradable regardless of sole cropping or intercropping (Fig 2h and 2*i*).

## Discussion

In the current study, one of the significant results was that the intercropping of alfalfa with corn-rye resulted in the highest DM yield. This finding suggested a preferable forage potential relative to crop rotation or sole cropping. However, the CP and NDF contents of the same crop of either intercropping or sole cropping were similar, whereas CP and NDF yields significantly differed among different treatments. Thus, nutrient yields responded to the cultivation patterns stemmed from the difference in DM yield rather than the nutrient content. These results are consistent with those in previous studies which revealed that forage yields of intercropping systems involving clover (*Trifolium repens* L.) and barley (*Hordeum vulgare* L.) [11], clover and ryegrass (*Lolium multiflorum* L.) [30], faba bean and barley [6,31] are greater than those of sole cropping.

Although, in the present study, the field experiment is based on only one year of harvest data, when considering the production performance, a previous 3-year cultivation experiment also showed the yield advantage for intercropping compared with sole crops [14,17]. Therefore, given the biomass yield, we conclude that the intercropping of alfalfa and corn-rye improved the forage production performance and utilization efficiency of arable land; this intercropping practice is a feasible forage production pattern.

It should be pointed out that the yield advantage in the present study was based on the sum of the DM of each component crop, rather than the yield of each companion crop. Since the main aims were to certify whether inclusion of legume (alfalfa) in the traditional cereals rotation system can improve the total biomass, nutrient balance of harvest material, and the overall nutrient utilization. In fact, the yield per unit growth land of component crops in intercropping system is higher than the same crop in sole cropping system (Table A in S1 File.).

The rate and extent of feedstuff degradation in rumen can be used as reference to evaluate utilization in the digestive tract. The non-degradable nutrients within 48 h are unlikely utilized by ruminants and excreted directly. In the present study, intercropping system provided more degradable nutrients in terms of DM, CP, and NDF than sole cropping or crops rotation. This finding suggested that available nutrient yields from the intercropping harvest mixed material consisting of legume and grasses were enhanced. Previous studies also documented that the combination of grass and legume hays used as livestock diet likely produce the positive associative effects with improved nutrient utilization that exceeds balanced median values of its components considered individually [32,33]. Similarly, under the current experimental condition, the harvest mixed material from intercropping system comprised cereals and legume hay. Therefore, the higher nutrient utilization may be derived from the positive associative effects.

In the present study, the intercropping harvest material composed of corn, rye and alfalfa exhibited a higher nutrient degradability compared to that of individual corn or rye. This was consistent with previous studies. Some researchers found that the legumes had a faster degradation in comparison with grasses, and the presence of legumes likely accelerate the degradation of the grasses [34]. In addition, the association of legume with grass can elicit a synergetic effect with improved effective degradability, which might come from the optimal energy-to-protein ratio and the increased microbial profiles due to the multiple substances [35]; on the other hand, the associative effects between grass and legumes may be attributed to those bioactive compounds contained in component forages[36]. Similarly, in the current study, the DM effective degradability (*E*) for the intercropping harvest mixed material was higher than that from rotation cereals, and lower than that for sole alfalfa. On the other hand, compared to those for rotation crops, the DM degradation parameters involving fraction ‘*a*’ (rapidly degradable), ‘*b*’ (slowly degradable), were higher, and lag time (*L* values) was shorter for intercropping harvest material. Therefore, it is seemed that the rate and extent of forage degradation were related to inherent feed characteristics as indicated by the degradation kinetics of the *a*, *b*, *c*, and *L*. This was consistent with previous literatures [37,38].

Although the fraction *E* of the degradable NDF was intermediate between sole crop rye and corn, the mixed sample exhibited a significantly higher degradability of DM and CP than the mono-cultured crops due to high levels of fractions ‘*a*’ (rapidly degradable) and ‘*c*’ (degradation rate). Consequently, for these nutrient fractions *E* from intercropping harvest material were superior to the rotation crop. Fraction ‘*d*’ of degradable nutrients were also significantly increased in the mixture subjected to intercropping with shorter lag time compared with that of rye or corn subjected to sole cropping.

In the current study, intercropping mixture and sole forage stalks exhibited differential degradability depending on the compositions of diets. Furthermore, the combination of legume and grass in the intercropping harvest material likely elicited a positive associative effect. Alfalfa contained the greatest rapidly degradable fractions of DM, CP, and NDF, and was also composed of completely lignified ring-like vascular bundles with the main components of NDF; as a result, DM and NDF remained in rumen for a longer lag time. Those factors contributed to a greater effective degradability (*E*), potential degradable fraction (*d*) of DM, CP and NDF of alfalfa than those of the stem of other crops subjected to sole crop or rotation. The intercropping mixture composed of alfalfa, corn (stalk and grain), and rye stems presented a variation tendency similar to that of alfalfa hay subjected to sole cropping in terms of nutrient digestibility with improved fractions of ‘*E*’ and ‘*d*’ of the mixture.

Thus, the combination of alfalfa with gramineae straws (e.g. corn stem and rye stem) used as ruminant diet likely improves nutrient degradability. Especially, for CP digestibility, inclusion of alfalfa hay showed a large-scale enhancement compared with grasses alone. Similarly, Niderkorn et al. (2011) indicated the association of legume and grass possibly caused a synergetic response with a positive effect on nutrient use by animals [36]. Some previous studies also observed the positive response when legumes and grasses are mixed as livestock diets [32,39]. However, few studies have assessed forage performance and nutrient values based on the food chain from forage field production to livestock utilization. This method can be used to comprehensively evaluate feed yield potential and quality.

In the present study, forage yield and nutrient degradability were determined and degradability was illustrated on the basis of anatomical structure variation of stem tissues. In the current *in vitro* trial, lignified alfalfa stem tissue were partially degraded, and only non-lignified tissues can be completely degraded when incubation time was extended (data not shown). The degradation extent and rate can be illustrated by tissue sections [22,40]. Jung and Casler (2006) demonstrated that for the corn stem being exposed to rumen microbes, the nearest parenchyma cell layers surrounding vascular bundles were completely degraded, whereas other parenchyma cells far from the vascular tissues were degraded to a less extent. In contrast, for alfalfa stem, parenchyma cells in the pith located in the center of the stem cross-section can be completely degraded by rumen microbes within 8 h of fermentation [22,24,41].

After the 48-h *in vitro* incubation of alfalfa stem subjected to sole cropping, parenchyma cells located in the interior of the xylem ring appeared to be unlikely degraded. However, for the mixed fermentation samples, only a ring xylem was remained. Accordingly, the mixture consisting of alfalfa, corn, and rye stems showed enhanced degradability compared with alfalfa stalk subjected to sole cropping. Similarly, Jung and Engels (2001) found that the lignified ring structure in alfalfa stem comprising primary and the secondary xylem vessels remained intact even after 96 h *in vitro* incubation with rumen microorganisms [22]. Thus, the lignified ring-like structure is the main factor that reduces the degradability of alfalfa hay in ruminants. More tissues are possibly degraded and the complete utilization is likely improved when alfalfa hay and grasses are degraded simultaneously.

## Conclusions

The results obtained in the present study showed that intercropping of alfalfa and corn-rye increased total forage and degradable nutrient yields. An increase in intercropping system nutrient could be attributed to the increase of forage DM rather than nutrient content.

The harvest materials from legume-cereal intercropping system likely elicited a positive associative effect when those materials were degraded in the rumen, and exhibited a greater effective degradability than stems of simple crop subjected to sole cropping. Tissues of the harvest material subjected to intercropping were degraded to a higher extent than those of stems of crops subjected to sole cropping when these tissues were exposed to rumen microbes, and this characteristic contributed to the improved degradability of the legume-cereal intercropping harvest materials.

The present study explored the nutrient yield and degradability of the practical harvest material from intercropping system (alfalfa/corn-rye), demonstrating the feasibility of extensively employed intercropping cultivation pattern as a forage supply source to ruminant in farming area, and providing a theoretical support for further popularizing this model in a wider range. In addition, further research is also needed to investigate the difference of degradability between the material from sole crops mixed together and the actual harvest mixture from intercropping system.

## Supporting Information

S1 File The yields (kg/ha) per unit growth land area of component in different cultivation patterns (Table A). (DOCX)
