# Supplementary material for: Legume-Cereal Intercropping Improves Forage Yield, Quality and Degradability
Source: PLoS One. 2015 Dec 16;10(12):e0144813. doi: 10.1371/journal.pone.0144813 (PMC4687681; doi:10.1371/journal.pone.0144813)
Supplement: S1 File — (DOCX) [file pone.0144813.s001.docx]

**Supporting information**

**S1 File. The yields (kg/ha) per unit growth land area of component in different cultivation patterns**

In the present study, three cultivation patterns were replicated 3 times using a randomized complete block design, including the intercropping of alfalfa/corn-rye, rotation of corn-rye, and sole crop alfalfa. The intercropping system was established in May 20, 2011 with the alfalfa: corn row ratio of 5:2 in a 23.4m^2^ plot. The inter-row spacing was 20 cm for alfalfa and 40 cm for corn. There was a 30-cm gap between alfalfa and corn (rye) strips. The rye was sowed after the corn was harvested in intercropping and sole cropping system. The intercropping plot was 6.5m long and 2 bandwidths across. Thus, the intercropping area ratios occupied by alfalfa and corn (rye) were 55.56% : 44.44%.

In the present study, the intercropping advantage as regards the biomass and nutrient yield compared to the rotation or sole crop alfalfa was based on the sum of the DM of each component crop (i.e. the total DM yield per unit intercropping land area), rather than the yield per unit growing land area of each crop. Since the main aims were to certify whether inclusion of legume (alfalfa) in the traditional cereals rotation system can improve the total biomass, nutrient balance of harvest material, and the overall nutrient utilization (by degradability study ). This study explored the potential of forage production of different cultivation pattern by comparing the biomass, nutrient, and degradable nutrient yield following nutrient flow biological chain, investigated the degradability by *in sacco* method, and inspected the degraded tissues by microscopy inspection.

In fact, the yield per unit growing land area of component crops in intercropping system is higher than the same crop in sole cropping system. Those yield data were listed as supporting information (Table 1).

**Table A**. Dry matter and nutrient yields (kg/ha) of each component crop per unit growth land area in different cultivation patterns

| Treatments | | DM Yield (kg/ha.) | Nutrient Yield (kg/ha) | |
| --- | --- | --- | --- | --- |
|  |  |  | CP | NDF |
| Intercropping | corn | 26895 | 2390 | 12212 |
|  | rye | 10689 | 1136 | 6863 |
|  | alfalfa | 31859 | 6159 | 11935 |
| Rotation | corn | 23471 | 2079 | 10657 |
|  | rye | 7656 | 808 | 4919 |
| Sole crop | alfalfa | 22428 | 4154 | 8402 |
| SEM |  | 134.6 | 34.5 | 58.6 |
| ANOVA |  |  |  |  |
| Main effects |  | <0.001 | <0.001 | <0.001 |
| Intercropping *vs*. | rotation |  |  |  |
|  | corn | <0.001 | <0.001 | <0.001 |
|  | rye | <0.001 | <0.001 | <0.001 |
| Intercropping *vs*. | sole crop alfalfa | <0.001 | 0.002 | <0.001 |
